# Realizing Single Chip White Light InGaN LED via Dual-Wavelength Multiple Quantum Wells

**DOI:** 10.3390/ma15113998

**Published:** 2022-06-03

**Authors:** Yangfeng Li, Cui Liu, Yuli Zhang, Yang Jiang, Xiaotao Hu, Yimeng Song, Zhaole Su, Haiqiang Jia, Wenxin Wang, Hong Chen

**Affiliations:** 1 Key Laboratory for Renewable Energy, Beijing Key Laboratory for New Energy Materials and Devices, Beijing National Laboratory for Condensed Matter Physics, Institute of Physics, Chinese Academy of Sciences, Beijing 100190, China; zhangali815432952@163.com (Y.Z.); hxtbilly2093@163.com (X.H.); 17853137315@163.com (Z.S.); mbe2@iphy.ac.cn (H.J.); wxwang@iphy.ac.cn (W.W.); 2 Center of Materials and Optoelectronics Engineering, University of Chinese Academy of Sciences, Beijing 100049, China; 3 Semiconductor Manufacturing International Corporation, Beijing 100176, China; skliucui@163.com; 4 Beijing Key Laboratory for Magneto-Photoelectrical Composite and Interface Science, School of Mathematics and Physics, University of Science and Technology Beijing, Beijing 100083, China; b20180340@xs.ustb.edu.cn; 5 Songshan Lake Materials Laboratory, Dongguan 523808, China

**Keywords:** InGaN LEDs, white light, single chip, CRI, CCT

## Abstract

Dual-wavelength multiple quantum wells (MQWs) have great potential in realizing high quality illumination, monolithic micro light-emitting diode (LED) displays and other related fields. Here, we demonstrate a single chip white light indium gallium nitride (InGaN) LED via the manipulation of the dual-wavelength MQWs. The MQWs contain four pairs of blue light-emitting MQWs and one pair of green light-emitting QW. The fabricated LED chips with nickel/gold (Ni/Au) as the current spreading layer emit white light with the injection current changing from 0.5 mA to 80 mA. The chromaticity coordinates of (0.3152, 0.329) closing to the white light location in the Commission International de I’Eclairage (CIE) 1931 chromaticity diagram are obtained under a 1 mA current injection with a color rendering index (CRI) Ra of 60 and correlated color temperature (CCT) of 6246 K. This strategy provides a promising route to realize high quality white light in a single chip, which will significantly simplify the production process of incumbent white light LEDs and promote the progress of high-quality illumination.

## 1. Introduction

Indium gallium nitride (InGaN) light-emitting diodes (LEDs) are essential for high quality illumination [1,2,3] and high resolution, high brightness and fast response displays [4,5,6], thus playing an important role in lighting, high definition TV (HDTV), visual reality (VR)/augmented reality (AR) displays, etc. [7,8]. The white light LEDs have already taken over from the incandescent bulbs as the mainstream illuminating sources. The most popular method to realize white light emission at present is the combination of blue InGaN LEDs with scattered yttrium aluminum garnet (YAG) phosphor, which will emit yellow light when excited by the blue light, thus mixing with the excitation light to render a white light from sight view. However, such a method has some limitations such as the low color rendering index (CRI), high correlated color temperature (CCT) and nonuniform colors with different sight view angles [9,10]. Combining three red, blue and green (RGB) LEDs together would significantly improve the CRI, and the light quality could be modulated as the three chips are pumped by different circuits. However, such a process is rather complicated. Therefore, realizing white light emission in a single chip becomes a possible candidate for avoiding such drawbacks. Wang et al. demonstrate a single-chip white light LED by inserting an underlying InGaN preparation layer to facilitate the fabrication of InGaN quantum dots (QDs), thus leading to two to three emission peaks which render a white light from sight view [10,11]. Other researchers report a white light emission through InGaN/GaN nanorods or truncated pyramid structures [12,13,14]. In addition, the optically pumped monolithically multi-color lasing is demonstrated from an InGaN microdisk structure [15]. Wang et al. fabricate a 3D InGaN/GaN MQWs LED structure, the colors of which could be tuned from red to blue [9]. With the aid of V-pits and a programmable driving power supply module, they demonstrate a single chip white light emission with a CRI value larger than 90. The aforementioned progresses are stirring and inspiring. However, it seems that to precisely control the emission light, one may encounter some difficulties which may hinder the application in the manufacturing production. A hybrid MQWs structure with dual/triple-wavelength may be a practical approach to controllably realize white light emission in a single chip. Hussein S. El-Ghoroury et al. demonstrate a full-color LED structure with three types of MQWs, i.e., blue-, green- and red-emitting MQWs, the color of which changes from red to blue when the injection current increases [16]. Although the electroluminescence (EL) is simply measured from the as-grown wafer rather than from the fabricated LED chips, the results reveal a promising application of realizing white light emission through hybrid MQWs. Recently, Khoury et al. reported a white light LED with the chromaticity coordinates of (0.37, 0.42) on (20–21) GaN/sapphire templates [17]. The active region contains a blue QW and a yellow QW, demonstrating the important role of dual-wavelength MQWs in the white light emission.

In this study, we propose a dual-wavelength InGaN MQWs LED structure with blue and green QWs heteroexpitaxially grown on patterned sapphire substrate (PSS). The structure of MQWs is investigated by high resolution X-ray diffraction (HRXRD) and transmission electron microscopy (TEM). Furthermore, the emission properties are studied by temperature-dependent photoluminescence (TDPL) and excitation power-dependent photoluminescence (PL) and EL. A white light emission is attained in a current injection range of 0.5 mA to 80 mA. The chromaticity coordinates are close to the white light location in the Commission International de I’Eclairage (CIE) 1931 chromaticity diagram, and the color rendering index (CRI) Ra reaches 60 under a 1 mA current injection.

## 2. Experiment

The dual-wavelength LED structure was grown on a 2-inch PSS by an AXITRON G3 2400HT metal organic chemical vapor deposition (MOCVD) system. At first, a 25 nm-thick nucleation layer was deposited, followed by a 2 μm-thick nominally undoped intrinsic GaN layer. Then, a 2 μm-thick silicon doped n-GaN was grown with the active region on it. The active region consisted of 4 pairs of blue MQWs (2.5 nm In_0.13_Ga_0.87_N/14 nm GaN) and 1 pair of green QW (3 nm In_0.2_Ga_0.8_N/14 nm GaN). Then, a 100 nm-thick p-GaN ended the expitaxial process. The as-grown wafer was post annealed in N_2_ ambient at 700 ℃ to activate the magnesium dopant. The MQWs structure was evaluated by HRXRD and TEM. The lamella for TEM was cut and thinned by the focused ion beam (FIB) with gallium ions, and before thinning, a 2.5 μm-thick platinum (Pt) was deposited on the surface for avoiding ion damage. The optical performance was investigated by the excitation power-dependent PL at 90 K with a 325 nm He-Cd laser as the excitation source, and the TDPL from 15 K to 300 K with the same excitation laser at an excitation power of 18 mW [18]. Then, part of the as-grown wafer was fabricated to 300 μm × 300 μm LED chips with the standard LED fabrication process, where 4 nm nickel/8 nm gold (Ni/Au) was adopted to act as the current spreading layer for p-GaN, and Cr/Ti/Al was employed to act as the contact electrodes for both p- and n-GaN [19,20]. The current-voltage (I–V) properties were measured by a Keithley 4200 machine, while the EL spectra under different current injection conditions were obtained by the WEIMING ZCLED-12A10 LED tester. The chromaticity coordinates, CRI and CCT were extracted from the EL spectra.

## 3. Results and Discussion

**Figure 1:**
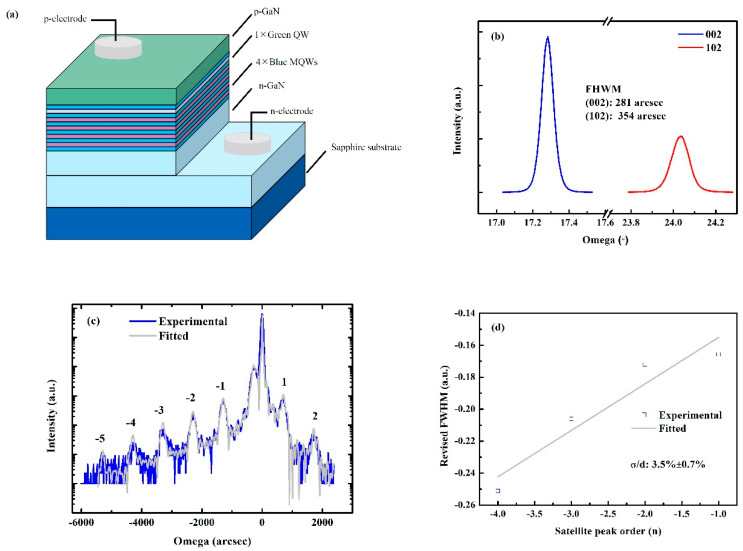

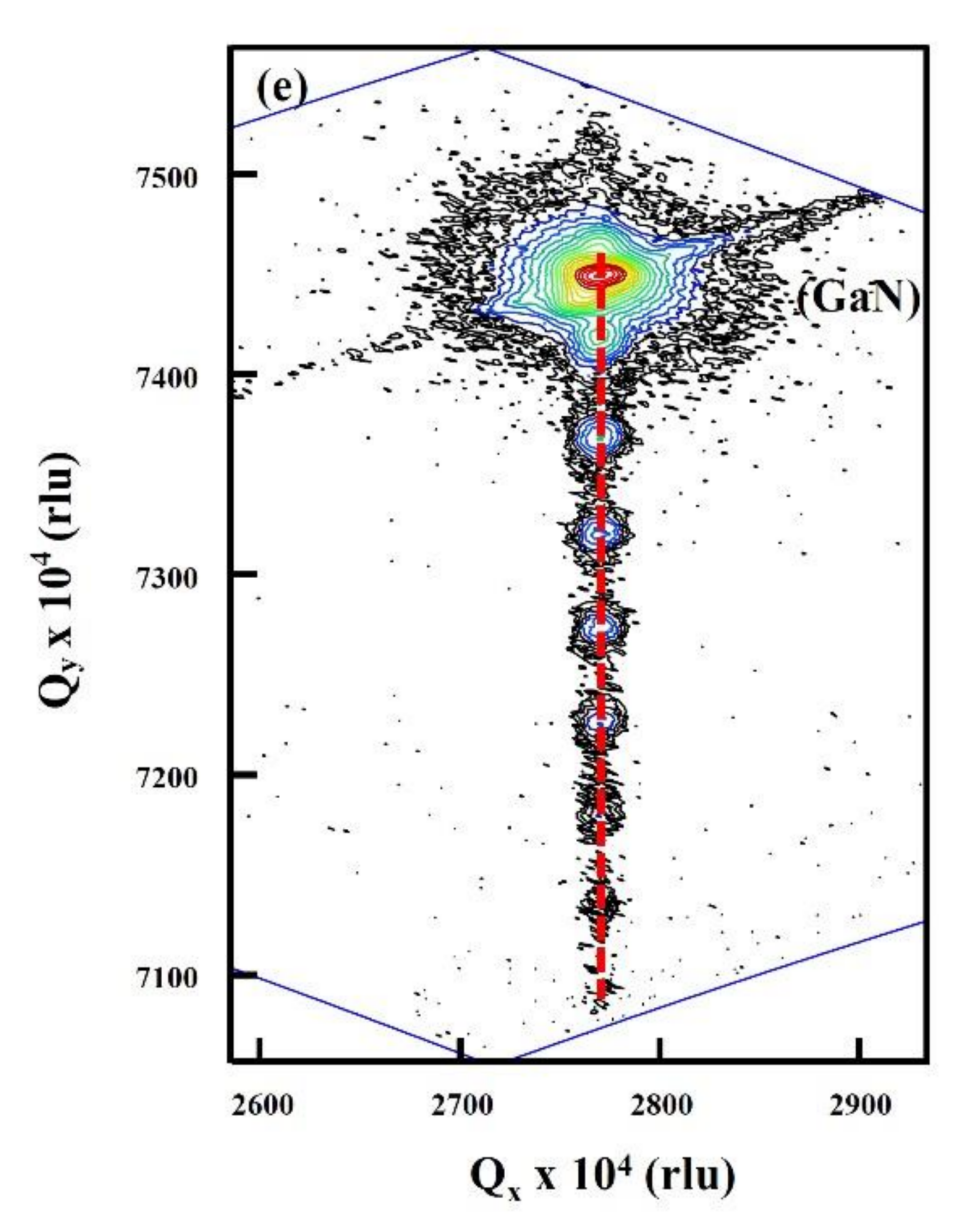
(**a**) The schematic illustration of LED structure. (**b**) The rocking curves of GaN (002) and (102) plane. (**c**) The ω-2 θ curve and the fitted line. (**d**) The revised FWHM of the satellite peaks. The light grey line is the fitted line. (**e**) The RSM image of the LED. The red dotted line is the guide line.

The LED structure is shown in Figure 1a. The rocking curves as well as the full-width at half-maximum (FWHM) values for GaN (002) and (102) planes are presented in Figure 1b. The relatively lower FWHM values indicate reduced dislocation densities both for screw and edge components [21,22]. Figure 1c delineates the omega-2 theta (ω-2 θ) curve of the as-grown sample with the light grey line as the fitted line. Up to “-5th” satellite peaks could be clearly distinguished. The fitted results are presented in Table 1, including the thicknesses of well and barrier as well as the indium composition for both blue and green MQWs. The fitted values coincide well with the designed parameters in the experimental part. The interface roughness of the MQWs is evaluated by the following equation [23]:

(1) $$\frac{w}{\Delta \theta_{M_{n}}}=\frac{2}{M}{\left(\frac{\ln2}{\pi }\right)}^{\frac{1}{2}}+\frac{{\left(\ln2\right)}^{\frac{1}{2}}\cdot \sigma \cdot n}{d}$$

where *w* is the FHWM of the nth satellite peak, *n* is the order of satellite peaks, Δ*θ_Mn_* is the space between adjacent satellite peaks, *M* is the number of periods of MQWs, *σ* represents the interface roughness of the MQWs and *d* is the period thickness of the MQWs. The deduced interface roughness value is listed in Table 1, which is comparable to other InGaN MQWs [24]. The reciprocal space mapping (RSM) of the as-grown sample is depicted in Figure 1e. It is obvious that the MQWs are totally strained to the GaN template [25,26].

**Figure 2:**
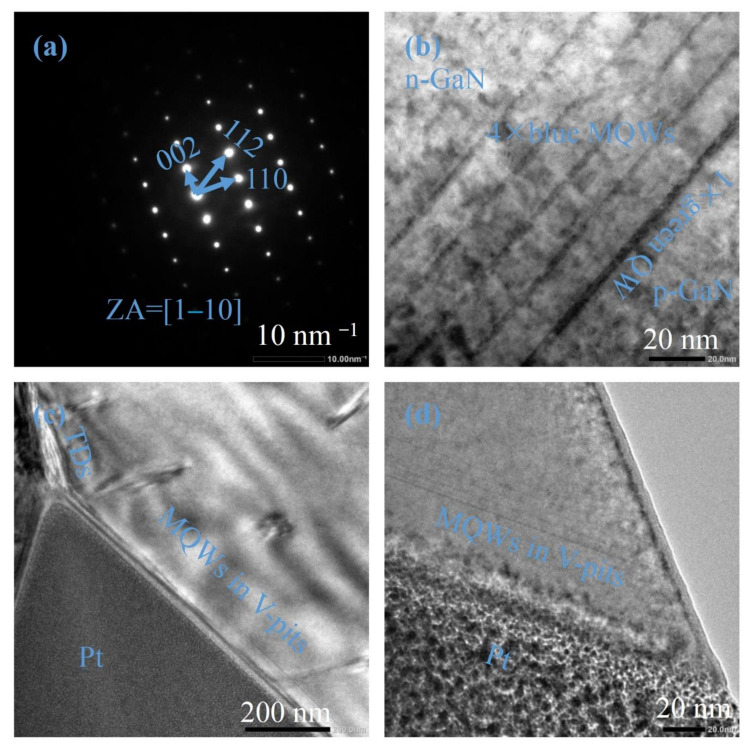
(**a**) The SADPs of the LED. (**b**) The MQWs of the LED. (**c**) The MQWs grown on the sidewall of a V-pit. (**d**) The enlarged image of the MQWs grown on the sidewall of a V-pit.

The LED structure is further confirmed by the TEM. The selected area diffraction patterns (SADPs) are represented in Figure 2a, where the zone axis (ZA) is along the [1,2,3,4,5,6,7,8,9,10] direction. The MQWs’ structure is shown in Figure 2b, where the 4× blue MQWs and 1× green QW are clear to be seen. The thicknesses of the blue QW, green QW and barrier measured from Figure 2b are 2.5 nm, 3.2 nm and 13.1 nm, respectively, consistent with the XRD results. Figure 2c demonstrates the MQWs grown on the sidewall of a V-pit. The MQWs are more slender than those in Figure 2b due to the slow growth rate on the semi-polar planes [27,28]. On the zenith of the V-pit, threading dislocations (TDs) emerge. Figure 2d is the enlarged image for the MQWs adjacent to a V-pit, where the well thicknesses for blue and green QWs and barrier thickness are measured to be 1.2 nm, 1.4 nm and 3.2 nm, respectively, reduced by a factor of more than two compared to those in Figure 2b.

**Figure 3:**
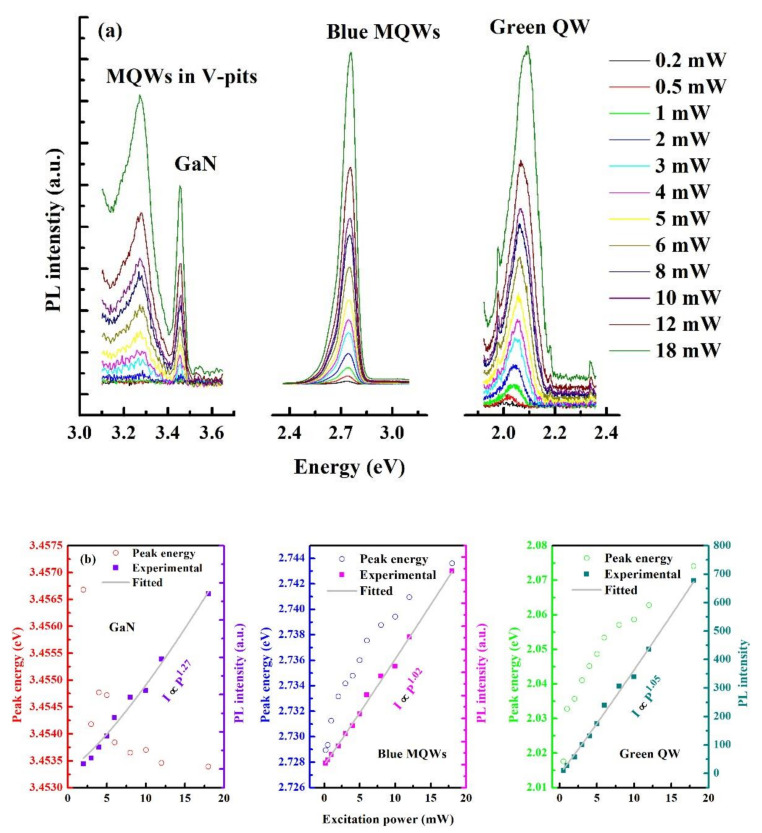
(**a**) The PL spectra of GaN, blue MQWs, green QW under different excitation powers. (**b**) The PL peak energy and integral intensity changing with excitation power for GaN, blue MQWs and green QW. The light grey lines are the fitted lines with a power-law function.

To investigate the optical performance of the as-grown sample, the excitation power-dependent PL was carried out at 90 K. The excitation power was changed from 0.2 mW to 18 mW by a neutral optical attenuator. The emission peaks for GaN, blue MQWs and the green QW are clearly discernable from the spectra (Figure 3a). The other peak locates at ~3.27 eV, which is between the GaN (3.45 eV) and blue MQWs (2.75 eV), and should be attributed to the emission from the MQWs on the sidewall of V-pits. As shown in Figure 2c,d, the MQWs on the sidewall of V-pits are more than half thinner than the normal MQWs; therefore, the emission peak should demonstrate a large blue-shift. Moreover, as the sidewalls of V-pits are semi-polar planes, where the indium incorporation efficiency is lower than the c-plane, the blue-shift of the emission peak will be exacerbated further, thus leading to the additional emission peak between GaN and blue MQWs [9,27]. The peaks are fitted by a Gaussian function, and the peak energies as well as the PL integral intensity for each peak are represented in Figure 3b. It is worth noting that the emission peak of GaN exhibits a red-shift as the excitation power increases, while the emission peaks for blue MQWs and the green QW are monotonously blue-shift. The red-shift for the GaN emission peak should be attributed to the bandgap shrinkage due to the heating effect introduced by the high excitation power. Furthermore, the blue-shift for blue MQWs and green QW should be due to the band filling effect. A higher excitation power will excite the photogenerated carriers to populate the higher energy bands, thus leading to a blue-shift of the emission peak. The carrier transport from barrier to well may also contribute to the blue-shift as suggested by Lu et al. in that the excitation source is within the nonresonant excitation regime [29]. The PL integral intensity changing with excitation power obeys a power law function, viz. $I\propto P^{m}$, where *I* is the PL integral intensity, *P* is the excitation power and *m* is the index [18,30,31]. The fitted results are demonstrated in Figure 3b. The index *m* of GaN is 1.27, which is larger than 1, indicating a Shockley–Read–Hall (SRH)-dominant recombination [30]. In addition, the m indices for blue MQWs and green QW are almost equal to 1, indicating the radiative recombination process is dominant, which benefits the high efficiency light emission [30].

**Figure 4:**
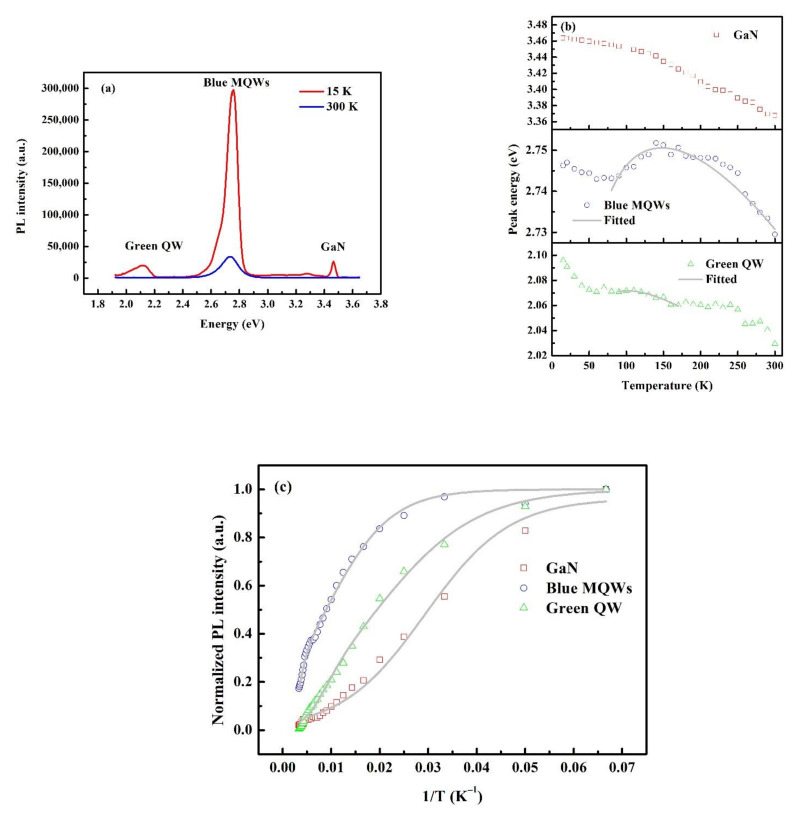
(**a**) The PL spectra at 15 K and 300 K. (**b**) The peak energy changes with temperature. The blue MQWs and green QW show an S-shaped curve, indicating the existing of localized states. The light grey lines are the fitted lines by the band-tail Varshni equation. (**c**) The normalized PL integral intensity vs. temperature. The light grey lines are the fitted lines.

To further verify the optical properties of the as-grown sample, TDPL is performed from 15 K to 300 K and the peak energy and integral intensity for each peak are extracted from the spectra. The spectra at 15 K and 300 K are shown in Figure 4a. At 15 K, the emission peaks of the green QW emission and GaN are prominent. However, such peaks are hardly to be distinguished when the temperature increases to 300 K. Furthermore, the emission from the blue MQWs dominates at all temperatures. Figure 4b demonstrates the evolution of peak energies of GaN, blue MQWs and green QW with temperature. For GaN, the emission peak red-shifts monotonously from 3.46 eV to 3.37 eV when the temperature ramps from 15 K to 300 K, coinciding with the bandgap shrinkage effect when the temperature increases [18]. However, the peak energies of blue MQWs and green QW exhibit an S-shaped curve when the temperature ranges from 15 K to 300 K due to the carrier transition from deep localized states to shallow localized states drifted by the thermal-activated energy. The S-shaped curves are fitted by the band-tail Varshni equation [3]:

(2) $$E(T)=E_{g}(0)-\frac{\alpha T^{2}}{T+\beta }-\frac{\Lambda^{2}}{kT}$$

where *E(T)* is the peak energy at a certain temperature *T*, *E_g_*(0) is the peak energy at 0 K, *α* and *β* are the fitting parameters, *k* is the Boltzmann constant and Λ is the degree of localized states. A higher value of Λ signifies a higher degree of localized states. The values of Λ for blue MQWs and green QW are 16.6 meV and 18.1 meV, respectively. The green QW preserves a relatively stronger localization effect. The PL integral intensity of all the three emission peaks decreases with the increment of temperature, indicating the activation of non-radiative recombination centers at higher temperatures. The ratio of the PL integral intensity at 300 K to that at 15 K is usually recognized as the internal quantum efficiency (IQE), with the assumption that all non-radiative recombination centers are frozen at a lower temperature (e.g., 15 K). Under such an assumption, the IQE values for GaN, blue MQWs and green QW are 2.0%, 17.3% and 0.5%, respectively. The green QW performs a lower IQE, which should be attributed to a higher non-radiative recombination effect. To further clarify the non-radiative recombination effect, we fitted the curves by the Arrhenius formulae. The formula for GaN is as follows [18]:

(3) $$I(T)=\frac{I(0)}{1+\alpha_{0}\exp(-\frac{E_{a}}{kT})}$$

where *I(T)* and *I*(0) are the PL integral intensities at temperatures *T* and 0 K, respectively; *α_0_* is a coefficient related to the defect density; *E_a_* is the thermal activation energy of a defect center; and *k* is the Boltzmann constant. The fitted curve is represented in Figure 4c and the fitted values are listed in Table 2, where α_0_ and *E_a_* are 29.6 and 10.0 meV, respectively. The activation energy of the non-radiative recombination center for GaN is in agreement with the results reported in other research works [18]. However, the relatively higher defect density renders the fast quenching of the emission when temperature increases, thus leading to a lower IQE (i.e., 2.0%). The formula for blue MQWs and green QW is as follows [3,32]:

(4) $$I(T)={\left[1+C_{1}\exp(-\frac{E_{A1}}{kT})+C_{2}\exp(-\frac{E_{A2}}{kT})\right]}^{-1}$$

where *I*(*T*) is the normalized integral PL intensity, *C*_1_ and *C*_2_ are two constants related to the density of non-radiative recombination centers, *E_A_*_1_ and *E_A_*_2_ are the activation energies of the shallow and deep non-radiative recombination centers and *k* is the Boltzmann constant. The fitted results are listed in Table 2. From Table 2, it is easy to find that the densities of non-radiative recombination centers for blue MQWs and green QW are comparable, while the activation energies for both the shallow and deep non-radiative recombination centers are larger in the blue MQWs than those in the green QW, especially the activation energy of deep non-radiative recombination centers, which is 2.5-fold higher in the blue MQWs than in the green QW. A higher activation energy means a lower probability to activate the non-radiative recombination centers; therefore, the high radiative efficiency is retained, thus leading to a higher IQE.

**Figure 5:**
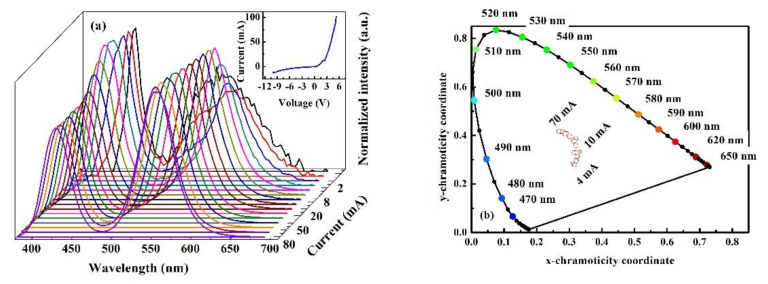
(**a**) The EL spectra from 0.5 mA to 80 mA. The inset is the I–V curve. (**b**) The chromaticity coordinates in the CIE1931 chromaticity diagram of the LED spectra under different current injection conditions. The empty red circles denote the chromaticity coordinates and the solid light grey circle indicates the white light location.

The EL performance of the LED chips is investigated by undergoing different current injections. The EL spectra from 0.5 mA to 80 mA are illustrated in Figure 5a, with the intensity normalized to the highest peak value for each spectrum. From Figure 5a, one could easily distinguish two emission peaks (i.e., the blue peak ~430 nm and the green peak ~568 nm) with comparable intensities. The peak intensity of the blue emission is a little stronger when the current is lower than 6 mA, while the green peak gradually surpasses the blue one as the current increases. Such a phenomenon should be attributed to the facilitation of deep hole injection assisted by the large V-pits, as shown in Figure 2c,d. The V-pits make the current bypass the top QWs and directly inject into the deeper QWs [33,34]. With the ascending current, the bypass effect is weakened, hence leading to the enhancement of the top QWs’ emission, i.e., the green emission. The inset in Figure 5a shows the I-V curve for an LED chip, where the forward voltage at 20 mA is 2.85 V. The chromaticity coordinates (x, y) in the CIE1931 chromaticity diagram as well as the CRI and CCT at different injection currents are listed in Table 3. The (x, y) coordinates are also delineated in Figure 5b, showing a circumvention around the white light location. The CIE (x, y) coordinates corroborate the white light emission of the LED under different currents [17]. In addition, the CRI and CCT are also important indicators to evaluate the quality of the white light. A higher CRI value indicates a better color rendering property of the white light. In other words, the white light spectrum is closer to the sunlight or blackbody radiator. The CRI values based on eight reference objects (Ra) at different currents are calculated from the EL spectra. As the CCT values are all above 5000 K, a daylight illuminant is used in the CRI calculation [35]. The CRI value reaches 60 when the injection current is 1 mA with the CCT of 6426 K. It is a high value in the dual-wavelength white light emissions, as a high CRI value usually needs RGB three emissions. Only two emission peaks (i.e., blue and green) with the lack of red emission have the intrinsic drawback for realizing high CRI values. However, if we add a red emission QW into the active region, assisted by tuning the injection current, a CRI value larger than 90 should be anticipated, which should be investigated in the future.

## 4. Conclusions

We demonstrated a single chip white light LED with the CRI reaching 60 by dual-wavelength MQWs in this study. The white light property retains as the current, changing from 0.5 mA to 80 mA, with the chromaticity coordinates surrounding the white light location in the CIE1931 chromaticity diagram. This research indicates the potential of dual/triple-wavelength MQWs in realizing single chip white light LEDs, which will significantly simplify the hitherto white light LED process and render high-quality white light illuminants.

## Figures and Tables

**Table 1:** The structural parameters of the dual-wavelength LED.

	Blue MQWs			Green QW			$\frac{\sigma }{d}\left(\%\right)$
	Well/nm	Barrier/nm	Indium Composition/%	Well/nm	Barrier/nm	Indium Composition/%	
LED	3.0 ± 0.1	13.3 ± 0.1	13.7 ± 0.2	3.5 ± 0.2	13.9 ± 0.2	20.2 ± 1	3.5 ± 0.7

**Table 2:** The optical parameters: IQE; localization energy (Λ); activation energies (*E_a_, E_A_*_1_ and *E_A_*_2_); defect densities (α_0_, *C*_1_, and *C*_2_).

	IQE/%	Λ/meV	*E_a_*/meV	*E_A_*_1_/meV	*E_A_*_2_/meV	α_0_	*C*_1_	*C*_2_
GaN	2.0	-	10.0	-	-	29.6	-	-
Blue MQWs	17.3	16.6	-	13.0	103.4	-	3.8	136.5
Green QW	0.5	18.1	-	8.4	41.2	-	6.5	128.9

**Table 3:** The chromaticity coordinates (x, y), CRI and CCT at different currents.

Current/mA	x	y	CRI	CCT/K
0.5	0.3197	0.3862	49	5060
1	0.3152	0.329	60	6426
2	0.3118	0.2828	57	8652
4	0.3167	0.2784	45	8691
6	0.3215	0.2958	37	7481
8	0.3239	0.314	32	6661
10	0.324	0.3298	29	6153
15	0.3204	0.358	23	5568
20	0.3147	0.3768	21	5333
30	0.302	0.395	19	5255
40	0.2919	0.4072	18	5255
50	0.283	0.4136	17	5294
60	0.2753	0.4167	17	5411
70	0.2685	0.4177	18	5529
80	0.2872	0.407	18	5333

## Data Availability

The data that support the findings of this study are available from the corresponding author upon reasonable request.
